# The microvascular endothelial glycocalyx: An additional piece of the puzzle in veterinary medicine

**DOI:** 10.1016/j.tvjl.2022.105843

**Published:** 2022-07

**Authors:** Sara J. Lawrence-Mills, David Hughes, Melanie J. Hezzell, Matthew Butler, Chris Neal, Rebecca R. Foster, Gavin I. Welsh, Natalie Finch

**Affiliations:** a Bristol Renal, Bristol Medical School, University of Bristol, Bristol, UK; b current affiliation The Royal Veterinary College, University of London, North Mimms, UK; c Bristol Veterinary School, University of Bristol, Langford, UK; d Langford Vets, Langford House, Langford, UK

**Keywords:** Detection, Diagnosis, Disease, Physiology, Visualisation

## Abstract

The endothelial glycocalyx (eGlx) is a critically important structure lining the luminal surface of endothelial cells. There is increasing evidence, in human patients and animal models, for its crucial role in the maintenance of health. Moreover, its damage is associated with the pathogenesis of multiple disease states. This review provides readers with an overview of the eGlx; summarising its structure, essential functions, and evidence for its role in disease. We highlight the lack of studies regarding the eGlx in cats and dogs, particularly in naturally occurring diseases. Importantly, we discuss techniques to aid its study, which can be applied to veterinary species. Finally, we present targeted therapies aimed at preserving, and in some cases, restoring damaged eGlx.

## Highlights

• The endothelial glycocalyx is critical in maintaining vascular health.
• Glycocalyx visualisation and measurement is technically challenging.
• There is growing evidence for the role of glycocalyx breakdown in human disease.
• Research is needed to understand glycocalyx degradation in disease in dogs and cats.

## Introduction

The glycocalyx (Glx) is a gel-like matrix comprised of a combination of glycosaminoglycan (GAG) sugar chains, proteins, and enzymes, arranged in a mesh work. The most commonly found molecules are glycoproteins and proteoglycans. The Glx is a microscopic structure coating the extracellular membrane of many living cells, including epithelial cells and some bacteria. This review will focus on the endothelial Glx (eGlx) covering the luminal surface of the endothelial cells. The eGlx plays a critical role in the maintenance of health and its perturbation contributes to the pathogenesis of a plethora of diseases. Increased understanding of the eGlx and its contribution to vascular permeability has led to the revision of Starling’s principle (Jacob et al., 2007). The eGlx is increasingly recognised as a therapeutic target with the potential to slow the progression of many diseases. Yet, in veterinary species its importance has largely gone unrecognised, likely due to the challenges associated with its study.

## Glx structure

**Fig. 1:**
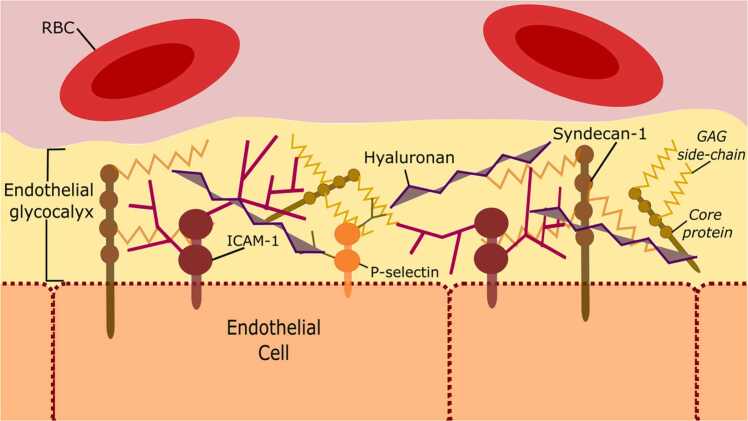
Schematic representation of the key structural components of the endothelial glycocalyx. GAG, glycosaminoglycan; ICAM-1, intercellular adhesion molecule-1; RBC, red blood cell.

The basic structure of the Glx is conserved between prokaryotic cells, including bacteria, and eukaryotic cells, including epithelial and endothelial cells. The major components are summarised in Fig. 1. Proteoglycans, forming the eGlx backbone, are composed of core proteins and covalently bonded, negatively charged, unbranched GAG side chains. Proteoglycans vary in their number and type of side chains, size of core proteins and their extent of membrane binding. The GAG side chains are composed of linear polymers of disaccharides varying in length and modification, such as acetylation and sulfation. The GAG, hyaluronan, binds directly to the endothelial cell membrane via cell surface receptors (Smart, 2016). There are numerous glycoproteins contained within the eGlx, they are characterised by small-branched carbohydrate chains, examples include endothelial cell adhesion molecules and components of the coagulation and fibrinolytic cascade. The expression of glycoproteins is highly variable, depending on endothelial cell activation (Jung and Ley, 1997). This multi-layered structure also contains various soluble components such as receptors (e.g. fibroblast growth factor receptor (Gloe et al., 2002)), enzymes, and growth factors. Other plasma derived molecules, including albumin, transiently bind to components of the eGlx (Jacob et al., 2007). The richly diverse composition of the eGlx is in dynamic equilibrium with blood, its structure changing in response to the microenvironment.

Significant inter-species differences in eGlx structure are inferred from techniques such as lectin staining. For example, differing staining patterns in mouse and rat kidneys with lectin conjugates have been demonstrated (Schulte and Spicer, 1983). Inter-species differences in eGlx structure may also contribute to variable eGlx damage following exposure to harmful stimuli, a potential explanation for unique species susceptibility to vascular disease. For example, atherosclerosis, in which eGlx damage contributes to the formation of atherosclerotic plaques (van den Berg et al., 2006, Cancel et al., 2016), represents a significant cause of morbidity and mortality in humans whilst cats and dogs appear naturally resistant (Heron and Anderson, 2016, Seidelmann et al., 2018). EGlx structure may also vary between sexes. This is suggested by the finding that female patients with coronary artery disease had a greatly reduced eGlx depth compared to healthy controls, as a result of greater eGlx shedding in response to disease (Brands et al., 2020), while no difference was found in male patients. Comparative studies exploring the unique differences in eGlx structure between species, sexes, or even individual breeds are yet to be performed. Future research should consider these potential confounding factors in their study design.

## Visualisation of the eGlx

There are several methods for eGlx detection and visualisation, which are summarised in Table 1. Due to the extreme fragility and small depth of the eGlx, visualisation is challenging. The eGlx is easily disturbed, dehydrated, and consequently lost during many preparation protocols resulting in many studies underestimating its depth (Luft, 1966, Ueda et al., 2004).

**Table 1:** Advantages and disadvantages of the key glycocalyx detection methods.

	Technique	Advantages	Disadvantages
Direct Detection	Perfusion fixation and staining with heavy metal ions	Reference standard technique	Technically challenging; clinically impractical
	Immersion fixation and staining with heavy metal ions	Quick; simple	Variable success; requires tissue biopsy
	Immersion fixation and lectin staining	Simpler sample collection and processing than perfusion techniques	Species- and organ-specific; ‘peak-to-peak’ technically challenging
Indirect Detection	Quantification of circulating eGlx components (ELISAs)	Clinically applicable; minimally invasive	Applicable to specific components
	Sidestream dark field imaging	Clinically applicable; non-invasive	eGlx depth inferred; only suitable for anaesthetised animals

eGlx, endothelial glycocalyx; ELISA, enzyme linked immunosorbent assay.

### Direct visualisation techniques

#### Perfusion fixation

**Fig. 2:**
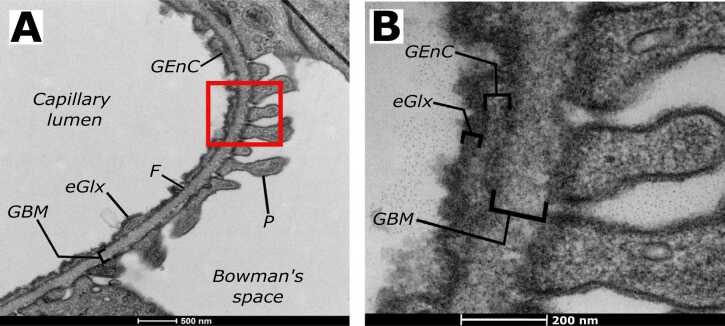
Electron micrograph images using Alcian blue staining of the endothelial glycocalyx (eGlx) on the surface of rat glomerular endothelial cells. The red box indicates the area in (A) magnified in (B). F, endothelial fenestration; GBM, glomerular basement membrane; GEnC, glomerular endothelial cell; P, podocyte foot process.

The most established and successful technique for direct visualisation of the eGlx involves vessel perfusion and staining (Hegermann et al., 2016). Initial vessel cannulation is required prior to flushing with solutions of physiological oncotic and electrolyte properties (Kimura et al., 2001, Oliveira et al., 2014) to remove plasma proteins and prevent eGlx dehydration (Jacob et al., 2007, Ebong et al., 2011). Following this, vessels are prepared for electron microscopy by perfusion with fixative solutions such as glutaraldehyde in cacodylate buffer prior to perfusion with special stains that bind heavy metal cations to proteoglycans within the eGlx (Curran et al., 1965, van den Berg et al., 2003). Fig. 2 demonstrates the binding of Alcian blue, arguably the most cited staining agent (van den Berg et al., 2003, Ebong et al., 2011), to sulfated GAG side chains present on proteoglycans in rat glomerular endothelial cells. Using this standard technique, the eGlx has been visualised in uterine and testicular arteries from dogs (Lawrence-Mills et al., 2022a, this issue). The method is technically challenging; vessels require flushing immediately post collection and at physiological pressures to prevent eGlx damage, further fixative solutions require the use of a fume hood. Perfusion fixation also lacks reproducibility both within and between studies, perhaps associated with its technical challenges, leading to discrepancies between eGlx measurements (Hegermann et al., 2016) and limiting the interpretation of these studies. For these reasons, this technique is not well suited to clinical samples.

#### Immersion fixation

The difficult and time-consuming nature of vessel perfusion has led to the development of immersion fixation methods. Some studies report excellent success with this technique, demonstrating images of similar quality to perfusion fixed samples (Yang et al., 2014). However, potential limitations include the spontaneous shedding following immersion (Chappell et al., 2009); poor penetration of cations makes the eGlx more prone to collapse upon fixation. Alternative studies have implemented immersion fixation prior to staining with lectins (Mulivor and Lipowsky, 2004) or specific fluorescent-labelled antibodies targeting eGlx components such as heparan sulfate (Florian et al., 2003). Few studies have compared a variety of eGlx fixation and visualisation techniques (Betteridge et al., 2017); however, the duplicity of sex-organ tissue does lend itself to such investigations.

#### Lectin staining

Lectins are glycoproteins isolated from plants, animals, bacteria, and viruses (Sharon and Lis, 2004). Uniquely, lectins bind specific carbohydrates and can be used to target GAGs within the eGlx (Mulivor and Lipowsky, 2004, Dane et al., 2013). A novel technique for eGlx quantification using lectin labelling termed ‘peak-to-peak measurement’, utilises cell membrane labelling with octadecyl rhodamine B chloride (R18) in combination with eGlx labelling lectin to derive a measure of the eGlx thickness (Betteridge et al., 2017). In vivo rat studies have demonstrated this peak-to-peak method correlates with eGlx measurements taken from electron micrographs (Betteridge et al., 2017). Others have also utilised lectin staining to study the eGlx in dogs (Meyer et al., 2008, Yabuki et al., 2012) and cats (Castagnaro et al., 1987, Roussel and Dalion, 1988, Kamiya et al., 1991). It must be noted that none of the aforementioned studies confirmed eGlx staining but rather assumed the staining to be associated with eGlx based on the location relative to the endothelium. Confirmation of eGlx specific staining requires either co-staining of the cell membrane, with R18 for example (Oltean et al., 2015), or further electron microscopy imaging (Mennander et al., 2012). Lectins also display different binding patterns in different studies. For example, wheat germ agglutinin (WGA) was reported to successfully label eGlx in feline heart muscle and brain in one study (Castagnaro et al., 1987) but not in another (Roussel and Dalion, 1988). The reasons for such differences are unclear but may include differences in succinylation of lectins, the use of different fixatives, variation in glycans expressed by individual animals, and variations in disease status.

### Indirect eGlx measurement

Many direct detection and visualisation techniques are unsuited for eGlx study in clinical samples. The quantification of circulating eGlx breakdown products in blood and urine is a more clinically applicable tool (Schmidt et al., 2016, Smart, 2016). Such methods that quantify eGlx components in biological fluids include colorimetric methods, high-performance liquid chromatography, mass spectrometry, and enzyme-linked immunosorbent assays (ELISA) (Imanari et al., 1992, Padberg et al., 2014, Schmidt et al., 2016). ELISAs can quantify specific eGlx components, for example hyaluronan, and syndecan-1 (Padberg et al., 2014, Yini et al., 2015). As discussed above, there can be species differences in eGlx structure related primarily to the arrangement and contribution of differing proteoglycans and glycoproteins (Dore et al., 1998). Therefore, specific assays may be more effective for the detection of eGlx shedding in some species than in others. The plethora of functions of hyaluronan have influenced its high conservation across species (Garantziotis and Savani, 2019), enabling cross-species use of hyaluronan ELISAs. Multiple studies in both humans and animal models have demonstrated a correlation between circulating components of eGlx and other direct and indirect measurements of eGlx thickness (Rehm et al., 2007; Vlahu et al., 2012). Therefore, the quantification of circulating eGlx components is widely accepted as a marker of eGlx health.

The use of ELISAs to measure eGlx components has been reported in dogs (Yini et al., 2015). Syndecan-1 and heparan sulfate ELISAs were used to evaluate the effectiveness of unfractionated heparin in protecting the eGlx in a canine model of septic shock (Yini et al., 2015). However, it is unclear whether these assays were validated for use in dogs. Measurement of hyaluronan using a commercially available ELISA validated for use with serum samples from dogs suggested eGlx shedding following rapid crystalloid fluid administration in a canine model of haemorrhagic shock (Smart et al., 2018). Plasma hyaluronan concentrations have been measured in healthy dogs to investigate the impact of varying fluid rates and demonstrated no significant differences in hyaluronan concentrations between fluid administration rates (Beiseigel et al., 2021). Recently, using the same hyaluronan ELISA, eGlx damage was demonstrated in dogs with naturally occurring myxomatous mitral valve disease and dogs in a hypercoagulable state (Lawrence-Mills et al., 2022b, this issue). Hyaluronan specifically has also been measured as a marker of vascular health in other diseases. For example, serum hyaluronan has been reported to be higher in dogs with portosystemic shunts (Seki et al., 2010). While serum hyaluronan concentration was shown to decrease from one month post shunt ligation (Devriendt et al., 2021). These studies used hyaluronan as a marker of liver perfusion due to its rapid catabolism by hepatic sinusoidal endothelial cells; however, the increased circulating concentrations could alternatively indicate eGlx damage in these diseases.

**Fig. 3:**
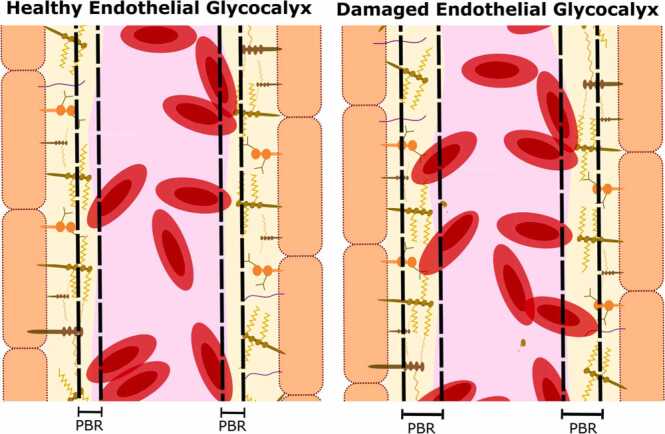
Schematic representation of the perfused boundary region (PBR), demonstrating two vessels, the left with a healthy endothelial glycocalyx and the right with a damaged endothelial glycocalyx.

The need for more non-invasive methods of eGlx measurement has led to the development of a technique that utilises sidestream dark field imaging of microvasculature (typically of the sublingual mucosa) in combination with specialist software. This technique infers eGlx depth by measuring the perfused boundary region (PBR). This is the intra-luminal area where flowing red blood cells can move through the eGlx and is distinct from the impermeable portion of eGlx (Fig. 3). Damage to the eGlx results in an increased PBR, thus PBR is an indirect measure of eGlx health (Martens et al., 2013). This technique is already being used in human patients to investigate the eGlx. For example, Pouska et al. (2018) used the PBR to investigate the impact of a fluid challenge on the eGlx and were able to demonstrate a significantly increased PBR following administration of 500 mL balanced crystalloid. Sidestream dark field imaging has been explored for veterinary use. Two studies have applied this methodology in a non-invasive way to measure the eGlx in the sublingual microvasculature of anaesthetised dogs (Londoño et al., 2018) and cats (Yozova et al., 2021). In horses, sidestream dark field video microscopy has been used to assess microvascular perfusion (Mansour et al., 2021), but not the eGlx. This technique has also been implemented to assess bowel microcirculation. In human patients; measurements of microvascular flow index have been shown to be similar between bowel and sublingual microvasculature (de Bruin et al., 2016). In veterinary species, sidestream dark field video microscopic assessment of jejunal eGlx has been proposed as a marker of jejunal microvascular health in dogs (Mullen et al., 2020), with potential applications that include aiding in the identification of compromised intestine requiring resection. Measurements in all studies in veterinary species were reported to be similar to those obtained in humans. However, findings were not correlated to any other direct or indirect measurement of the eGlx. Further, the requirement for access to sublingual or jejunal microvasculature and the need to obtain images over several minutes means at present this technique can only be used in anaesthetised animals.

## eGlx function

**Fig. 4:**
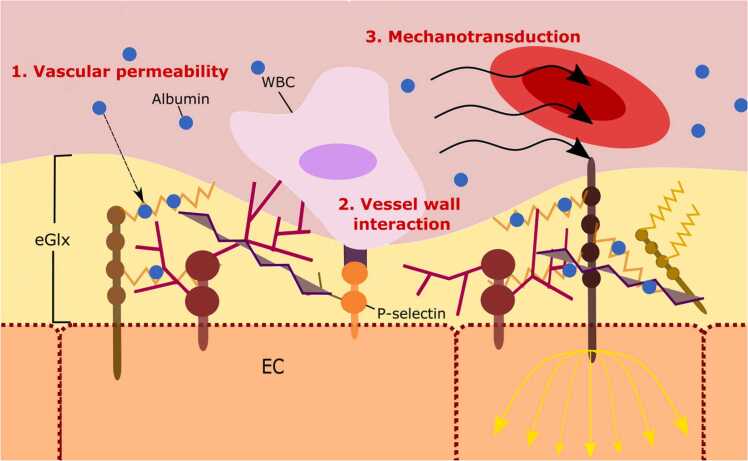
Main functions of the endothelial glycocalyx (eGlx). Vascular permeability is demonstrated by the exclusion of albumin. Vessel wall interaction is shown by the thin area of the eGlx exposing P-selectin on the endothelial membrane enabling leukocyte binding. Forces of plasma flow, depicted by the black arrows, are transmitted through the brown proteoglycan into the endothelial cell, indicated by the yellow arrows. EC, endothelial cell; WBC, white blood cell.

The eGlx contributes to vascular health through a multitude of functions. The primary functions are outlined in Fig. 4.

### Vascular permeability

The eGlx is critical to maintaining vascular homeostasis and overall health. It directly regulates vascular permeability because the size and spatial arrangement of proteins and disaccharides form a physical mesh and the heavily sulfated GAGs generate a net negative charge. The combination of physical and electrostatic properties of the eGlx create a macromolecular sieve excluding molecules > 70 kDa from reaching luminal endothelial cell membranes (Vink and Duling, 1996). Furthermore, binding of circulating albumin to the eGlx generates an oncotic pressure within the eGlx, thus locally increasing the intravascular oncotic pressure. Vascular permeability is therefore influenced by the oncotic pressures within the vessel lumen, the eGlx, and the interstitium (Jacob et al., 2007). Fluid movement across the vessel wall to the surrounding tissue is therefore determined by the difference between the oncotic pressures in all three compartments (Jacob et al., 2007, Salmon et al., 2009). This revises the traditional Starling’s force theory that previously only considered the global intravascular oncotic pressure relative to the surrounding tissue (Jacob et al., 2007, Smart, 2016).

### Vessel wall interaction

The eGlx regulates interactions between cells within the vessel, such as red blood cells and platelets, and the endothelial cell membrane. It provides a physical coating on the surface of the endothelial cells thus preventing direct contact with circulating cells (Pries et al., 1998). Furthermore, the ‘hiding’ of functionally active glycoproteins, such as selectins and integrins, helps to maintain vascular homeostasis. Degradation of the eGlx exposes intracellular adhesion molecule-1 resulting in leukocyte adherence and activation (Mulivor and Lipowsky, 2009). The fine balance between a hyper- and hypo-coagulable state is maintained through the storing of enzymatic cofactors within the eGlx, including antithrombin and tissue-type plasminogen activator (Johansson et al., 2011). The ability of the eGlx to bind enzymes such as superoxide dismutase also acts to protect the endothelium (Li et al., 1998). EGlx bound superoxide dismutase scavenges oxygen free radicals, thereby reducing oxidative stress and preventing endothelial dysfunction.

### Mechanotransduction

The eGlx acts as a mechanotransducer, enabling endothelial cells to respond to the forces induced by plasma flow. Shear stress is transmitted via eGlx transmembrane proteins, thought to bind intracellularly to the endothelial cell cytoskeleton (Pries et al., 2000). Shear stress signals determine eGlx composition, with chronic shear stress exposure increasing glycosaminoglycan synthesis (Arisaka et al., 1995). Furthermore, flow mediated vasodilation is regulated by the eGlx. Increases in blood flow cause conformational changes within the eGlx triggering the release of nitric oxide (NO) (Mochizuki et al., 2003) which in turn mediates local vasodilation and increased vascular permeability (Santos-Parker et al., 2017).

## Evidence for eGlx involvement in disease

There is a growing body of evidence supporting the role of eGlx shedding in animal models of disease and naturally occurring disease in humans, however, few studies have examined this in cats and dogs. Table 2 summarises the evidence for eGlx degradation in human patients with naturally occurring disease and the limited evidence in cats and dogs. The majority of evidence for the role of eGlx damage is from experimental animal models and spontaneous diseases in humans and veterinary species for the following states:

**Table 2:** Outlining specific examples of evidence for endothelial glycocalyx shedding in different disease states in humans and veterinary species.

	Reference	Disease state	Marker used	Finding
Human patients	Schmidt et al. (2016)	Sepsis; ARDS	Urinary GAG fragment (HS, CS, and HA)	In septic patients, urinary GAG fragments correlated with kidney dysfunction, and urinary HS and HA concentrations were associated with mortality. In ARDS patients, higher urinary GAGs fragments were associated with risk of developing an AKI.
	Padberg et al. (2014)	CKD	Plasma syndecan-1 and HA	Plasma syndecan-1 and HA increased across CKD stages and increases were independently associated with kidney dysfunction.
	Dane et al. (2014)	ESRD	PBR; plasma syndecan-1	Compared to healthy controls, ESRD patients had increased PBR and higher plasma syndecan-1.
	Vlahu et al., 2012	Dialysis patients	PBR; serum syndecan-1 and HA	Compared to healthy controls, dialysis patients had increased PBR, serum syndecan-1, and serum HA.
	Steppan et al. (2011)	Sepsis; major abdominal surgery	Plasma syndecan-1 and HS	Surgical and septic patients had increased plasma syndecan-1 and HS (septic > surgical).
	Nelson et al. (2008)	Sepsis	Plasma syndecan-1	Plasma syndecan-1 increased in septic patients. Increases were correlated with increased risk of mortality.
Veterinary Species	Shaw et al. (2021)	Septic peritonitis	Serum HA	Serum HA increased on day 2 or 3 of hospitalization. Interleukin-6 was a predictor of serum HA.
	Lawrence-Mills et al. (2022b)	Hypercoagulability; MMVD	Plasma HA	Increased plasma HA was found in dogs with hypercoagulability as well as dogs with MMVD.
	Naseri et al. (2020)	Canine parvoviral enteritis	Serum ESM-1	Higher serum ESM-1 was found in non-survivors.
	Yini et al. (2015)	Septic shock model	Plasma syndecan-1 and HS	Increased plasma syndecan-1 and HS was found following injection with a lethal dose of *E. coli*.

AKI, acute kidney injury; ARDS, acute respiratory distress syndrome; CKD, chronic kidney disease; CS, chondroitin sulfate; ESM-1, endothelial cell-specific molecule-1; ESRD, end-stage renal disease; GAG, glycosaminoglycan; HA, hyaluronan; HS, heparan sulfate; MMVD, myxomatous mitral valve disease; PBR, perfused boundary region.

### Sepsis

Sepsis refers to the systemic inflammatory response to infection (Singer et al., 2016). Sepsis is a leading cause of death in critically ill cats and dogs (Babyak and Sharp, 2016, Kenney et al., 2010). The eGlx is pathophysiologically important in sepsis due to its important roles in vascular permeability and tone. Alterations in eGlx composition following an inflammatory insult is thought to be one of the earliest features of sepsis in humans (Martin et al., 2016, Steppan et al., 2011). Dogs with septic peritonitis have been shown to have increased hyaluronan concentrations on day 2 or 3 of hospitalisation compared to admission, these concentrations decreased during the recovery period (Shaw et al., 2021). The same study identified relationships between serum interleukin (IL)− 6 and hyaluronan concentrations, suggesting an association between inflammation and eGlx degradation. In dogs with parvoviral enteritis, circulating endothelial cell-specific molecule-1 concentrations were increased compared to controls (Naseri et al., 2020) suggesting endothelial cell injury. However, the eGlx components syndecan-1 and heparan sulfate, were not found to be significantly different between affected dogs and controls (Naseri et al., 2020). This may be the due to small sample size or the timing of sampling (samples were collected at admission only, relatively early in the disease process) or poor assay performance.

The role of eGlx damage in perpetuating inflammation and vascular compromise has been demonstrated (Annecke et al., 2011, Schmidt et al., 2012). EGLx damage induces capillary leakage, platelet aggregation, coagulation, and loss of vascular tone (Annecke et al., 2011, Desjardins and Duling, 1990, Johansson et al., 2011) as well as expression of adhesion molecules, such as intercellular adhesion molecule-1, enabling leukocyte extravasation (Meerschaert and Furie, 1995, Stulc et al., 2008). The important role of eGlx shedding in perpetuating inflammation, and further eGlx damage, is evidenced by endotoxaemia induced eGlx shedding in mice initiating the activation of endothelial heparanase, a heparan sulfate-specific glucuronidase which induces eGlx degradation and neutrophil adhesion (Schmidt et al., 2012). The same study demonstrated heparanase inhibition attenuated sepsis-induced acute lung injury and mortality in mice.

EGlx shedding has been demonstrated to contribute to the pathophysiology of sepsis. Specifically, shed eGlx components have been demonstrated to act as damage-associated molecular patterns, further triggering proinflammatory cascades (Goodall et al., 2014). Heparan sulfate fragments have been shown to mediate cytokine release; the addition of soluble heparan sulfate to human blood mononuclear cells triggered the release of IL-1β, IL-6, IL-8, IL-10, and tumour necrosis factor (TNF), an effect mediated by Toll-like receptor-4 (Goodall et al., 2014). Further, the binding of heparan sulfate to interferon-gamma has been shown to significantly decrease its plasma clearance, as well as increasing its cytokine activity by 600%, through limiting the degradation of its carboxy-terminal domain (Lortat-Jacob et al., 1996). Moreover, eGlx degradation offers a plausible explanation for the hypercoagulable state of many septic patients (Hoppensteadt et al., 2015). Indeed, increased serum hyaluronan concentration is also reported in dogs with hypercoagulability (Lawrence-Mills et al., 2022b, this issue). The critical role of the eGlx in regulating haemostasis; including the regulation of interactions between circulating cells and the endothelial cell membrane (Pries et al., 1998), the ‘hiding’ of functionally active glycoproteins (Mulivor and Lipowsky, 2009, Schmidt et al., 2012), and the storage of enzymatic cofactors (Pries et al., 2000), makes eGlx shedding one of the plausible pathophysiological mechanisms contributing to hypercoagulability in dogs.

In addition to improved understanding of the pathophysiology of sepsis, knowledge of the effects of intravenous fluid administration on the eGlx may help prevent iatrogenic worsening of disease. Research in people has outlined the negative impact of over-infusion in septic patients (Kelm et al., 2015). Crucially, this is linked with increased mortality (Alsous et al., 2000, Uchino et al., 2006). These data have informed international guidelines for the treatment of sepsis, in which cautious use of fluid therapy is recommended following initial emergency resuscitation (Dellinger et al., 2008). Damage to the eGlx is considered to be one of the consequences of over-infusion (Chappell et al., 2014). In canine experimental models of haemorrhagic shock, rapid crystalloid administration increased eGlx shedding (Smart et al., 2018). This finding is in line with those in human patients and animal models and suggests avoidance of excessive fluid bolus adminsitration may be important in the management of shock in dogs. Markers of eGlx damage provide prognostic information in septic human patients (Kumar et al., 2006, Mouncey et al., 2015) and this may similarly hold true in dogs and cats. In human septic shock patients, circulating GAG concentrations were significantly increased in non-survivors compared to survivors (Nelson et al., 2008).

### Kidney disease, proteinuria, and microalbuminuria

Chronic kidney disease (CKD) is common in companion animals. One retrospective study identified 40 % of adult cats under 15 years of age had CKD, this increased to 80 % for cats above 15 years (Marino et al., 2014). One Swedish study reported a lifetime prevalence of CKD in dogs of 16 % (Pelander et al., 2015), although reports vary; some stating a prevalence as low as 3.7 % (O'Neill et al., 2013). In humans, systemic eGlx breakdown, quantified by circulating eGlx components, is correlated with kidney dysfunction (Dane et al., 2014, Vlahu et al., 2012b) with a 4-fold greater concentration of the eGlx components, syndecan-1 and hyaluronan, in CKD patients compared to healthy controls (Padberg et al., 2014). Further, incrementally higher concentrations of eGlx components are demonstrated in increasing stages of disease (Padberg et al., 2014). Additionally, the concentration of urinary GAG fragments in critically ill human patients correlates with kidney dysfunction (Schmidt et al., 2016). Studies evaluating eGlx damage and kidney function in cats and dogs are yet to be published; however, measurement of urinary GAG components offers a potential non-invasive biomarker. Moreover, improved knowledge of the role of the eGlx in chronic and acute kidney diseases in cats and dogs may allow development of much needed targeted therapeutics.

Systemic eGlx damage is reported in human CKD patients. In addition, eGlx damage specifically within the renal microvasculature can contribute to perturbations in renal permeability (Singh et al., 2007, Oltean et al., 2015, Desideri et al., 2018, Butler et al., 2019, Onions et al., 2019, Ramnath et al., 2020). In humans, glomerular eGlx shedding can occur following exposure to circulating inflammatory noxae (Padberg et al., 2014). Loss of glomerular eGlx integrity contributes to increased albumin (and potentially other macromolecule) permeability (Henry and Duling, 2000, Zdolsek et al., 2020). Thus, glomerular eGlx damage plays a role in the development of proteinuria and specifically albuminuria in glomerular injury (Singh et al., 2007, Lees et al., 2011). In vitro studies have supported the role of glomerular eGlx shedding in microalbuminuria in humans (Singh et al., 2007). Further, it has been hypothesised that microalbuminuria may signify systemic eGlx damage as well as glomerular eGlx damage (Singh and Satchell, 2011). In humans, microalbuminuria is clinically important as it is associated with increased risk of all-cause mortality (Xia et al., 2015), cardiovascular disease (Segura et al., 2004), progression of multiple aetiologies of kidney disease, and increased mortality due to kidney disease (Matsushita et al., 2010). The clinical significance of microalbuminuria in cats and dogs remains unclear, as does its relationship with glomerular or systemic eGlx damage.

### Hypervolaemia and hypertension

Hypervolaemia has been associated with increased mortality in hospitalised dogs (Cavanagh et al., 2016). Hypervolaemia and hypertension induce eGlx shedding in human patients and animal models (Ueno et al., 2004, Bruegger et al., 2011, Chappell et al., 2014). Hypervolaemia induced eGlx shedding in dogs is supported by findings of increased circulating hyaluronan following rapid large-volume crystalloid administration in an experimental canine haemorrhagic shock model (Smart et al., 2018). The same study investigated the impact of the type and rate, as well as overall volume, of fluid administered. Peak hyaluronan concentration was identified earlier, after 20 min, following isotonic crystalloid infusion, compared to later, after 40 min, following infusion of 4 % succinylated gelatin. Further, eGlx damage was greater with isotonic crystalloid at a high rate of infusion 80 mL/kg over 20 min, compared to other solutions at 20 mL/kg over 20 min. A different study has demonstrated increased plasma hyaluronan concentrations from baseline in dogs following both 5 and 10 mL/kg/h crystalloid fluid administration (Beiseigel et al., 2021), demonstrating even modest fluid rates have the potential to induce eGlx degradation. In human patients a significantly increased PBR has been demonstrated following a 500 mL crystalloid bolus in both spinal surgery patients and septic shock patients (Pouska et al., 2018). The PBR normalised within 60 min in spinal surgical, but not septic shock patients, suggesting that even conservative boluses may produce long-term eGlx damage in patients with an already vulnerable eGlx. Hypervolaemia in itself damages the eGlx via haemodilution of plasma proteins as well as osmotic alterations (Pries et al., 1998, Torres et al., 2013); however, it also induces the release of atrial natriuretic peptide from the atria of the heart (Chappell et al., 2014). Atrial natriuretic peptide induces eGlx shedding and loss of vascular barrier function in the coronary vascular bed of guinea pig hearts (Bruegger et al., 2005, Jacob et al., 2013).

The impact of hypertension on eGlx has been most extensively studied in brain endothelial cells. Early eGlx shedding, primarily in capillaries, was demonstrated in a spontaneously hypertensive rodent model, which compromised the integrity of the blood brain barrier (Ueno et al., 2004). In companion animals, systemic hypertension is most commonly a result of concurrent disease (Acierno et al., 2018). Up to 93 % of dogs with CKD (Anderson and Fisher, 1968), 67 % of dogs with diabetes (Marynissen et al., 2016) and 73 % of dogs with hyperadrenocorticism (Ortega et al., 1996) are hypertensive. Studies report 87 % of hyperthyroid cats and 46 % of cats with CKD to be hypertensive (Kobayashi et al., 1990). Uncontrolled systemic hypertension results in target organ damage within susceptible tissues including the kidney, eye, brain, and heart (Acierno et al., 2018). The pathophysiology of target organ damage is multifactorial; however, a key component is endothelial inflammation and injury (Schmid-Schönbein et al., 1991, McCarron et al., 1994, Schillaci et al., 2003). The vital role the eGlx plays in maintaining vascular integrity makes its involvement in target organ damage in human patients unsurprising, although this remains unstudied in cats and dogs. Importantly, restoration of the eGlx offers potential protection of important organs in patients with secondary hypertension, alongside anti-hypertensive medications and management of the primary disease.

## Therapeutic protection of the eGlx

Growing evidence for the contribution of eGlx damage in a plethora of human disease states has led to a focus in medical research into therapies to prevent eGlx shedding or restore damaged eGlx. Research into targeted therapies is in its infancy and there are currently no licensed products. To date, the evidence suggests that one of the most effective strategies for eGlx protection is by preventing fluid volume overload (Chappell et al., 2014). This has helped inform fluid therapy regimes in human patients (Dellinger et al., 2008) and cats and dogs (Byers, 2017). However, specific recommendations for fluid infusion rates remain heavily debated. In people with sepsis, rates of up to 30 mL/kg of crystalloid fluids over the first 3 h have been recommended (Rhodes et al., 2017). Updated guidelines grade the evidence behind this as low-quality and instead suggest the use of dynamic measures to guide fluid resuscitation (Evans et al., 2021). For example, monitoring changes in stroke volume in response to passive leg raise. However, species-specific blood volume and fluid tolerance limits direct extrapolations from recommendations for people to veterinary species. Instead, the consensus is for a patient-based individualised approach (Montealegre and Lyons, 2021). Isotonic crystalloids are generally the fluid type of choice in human and veterinary patients with colloids having the potential to result in a hypervolaemic state and eGlx damage (Rehm et al., 2001).

Alternatively, inhibition of enzymes that initiate eGlx degradation has been explored. Pre-treatment with etanercept, a TNF inhibitor, prevented an increase in plasma hyaluronan concentrations as well as decreasing coagulation activation in men injected with *E. coli* lipopolysaccharide endotoxin (Nieuwdorp et al., 2009). Doxycycline may stabilise the eGlx by inhibition of matrix metalloproteinases. Its administration to rats prior to and during insult decreased eGlx shedding, following infusion of artificial chemoattractant peptide f-Met-Leu-Phe into intestinal mesentery (Mulivor and Lipowsky, 2009). The clinical success of approaches that selectively inhibit eGlx degradation enzymes is likely to be limited due to the vast number of enzymes capable of damaging the eGlx, as well as the need to administer these inhibitors pre-emptively for maximal effect (Mulivor and Lipowsky, 2009, Purushothaman et al., 2010).

Reconstitution of the eGlx provides an additional therapeutic option. Sulodexide, a purified GAG containing 80 % heparan sulfate and 20 % dermatan sulfate (Veraldi et al., 2018), has demonstrated the most promise. Sulodexide is thought to promote eGlx regeneration, downregulate eGlx degradation enzymes and exert an anti-apoptotic and anti-inflammatory effect on endothelial cells (Ciszewicz et al., 2009, Broekhuizen et al., 2010). In human type-2 diabetic patients, sulodexide partially normalised vascular permeability postulated to be in part due to restoration of a healthy eGlx (Broekhuizen et al., 2010). However, larger clinical trials investigating the use of sulodexide in preventing progression of kidney and heart disease have proved inconclusive (Lewis et al., 2011, Packham et al., 2012), potentially due to study design. In dogs, the potential for eGlx reconstitution has been demonstrated in a canine model of septic shock in which unfractionated heparin infusion reduced eGlx shedding (Yini et al., 2015).

## Conclusions

The eGlx is a dynamic and fascinating structure with a critical role in health and disease. Composed of proteoglycans, glycoproteins, and soluble plasma proteins its composition is integrally linked to its function in regulating vascular permeability, endothelial cell interactions and mechanotransduction. Study of the eGlx is challenging. However, we have presented techniques in this review that are suited to veterinary clinical patients such as indirect measurement of eGlx components using ELISAs. In humans, there is much emerging evidence that eGlx damage prevents its normal function in maintaining vascular homeostasis and contributes to the pathophysiology and progression of many diseases. In veterinary species, research is in its infancy, although early studies have suggested eGlx degradation plays a role in naturally occurring disease in dogs. Research in veterinary species should initially focus on demonstrating the presence of eGlx damage in disease, and validating the tools necessary for its study, prior to evaluating potential therapeutics.

## Conflict of interest statement

This research was funded in whole, or in part, by the 10.13039/100004440Wellcome Trust (204813/Z/16/Z). None of the authors of this paper has any other financial or personal relationship with other people or organisations that could inappropriately influence or bias the content of the paper.
